# Deep mitigation for trade-embodied carbon emissions among the Belt and Road Initiative countries

**DOI:** 10.1016/j.isci.2024.110054

**Published:** 2024-05-22

**Authors:** Lina Zhang, Weichao Zhao, Yung-ho Chiu, Li Zhang, Zhen Shi, Changfeng Shi

**Affiliations:** 1 Business School, Hohai University, Nanjing 211100, China; 2 Department of Economics, Soochow University, Taipei 10048, Taiwan; 3 School of Mathematics, Hohai University, Nanjing 211100, China

**Keywords:** environmental management, environmental policy, global carbon cycle

## Abstract

The frequent trade within and beyond the Belt and Road Initiative (BRI) has prospered the economy but has also expanded carbon emissions. Here, through a multi-regional environmental input-output analysis framework, we explore the patterns and inter-sectoral linkage of trade-embodied carbon emissions among BRI countries during 2015–2019. Then, a dynamic data envelopment analysis model considering carbon inequality as a non-discretionary input is constructed to assess the carbon emission efficiency of the identified key sector. We find that trade-embodied carbon emissions in the BRI steadily increased during 2015–2019. The manufacturing sector was identified as the key sector, exhibiting an overall efficiency of 0.6268 on average, with significant efficiency disparities. Moreover, we validate the positive role of efficiency enhancement in carbon emission mitigation, as well as the negative moderating effect of carbon inequality. Overall, this study provides optimal collaboration and initiatives to mitigate trade-embodied carbon emissions among BRI countries deeply.

## Highlights

• Account for trade-embodied carbon emissions in the BRI between 2015 and 2019
• Identify the key priorities (i.e., countries and sectors) in the BRI
• Assess the carbon emissions efficiency of the identified key sector in the BRI
• Propose interventions to mitigate trade-embodied carbon emissions in the BRI

## Graphical abstract

**Figure:**
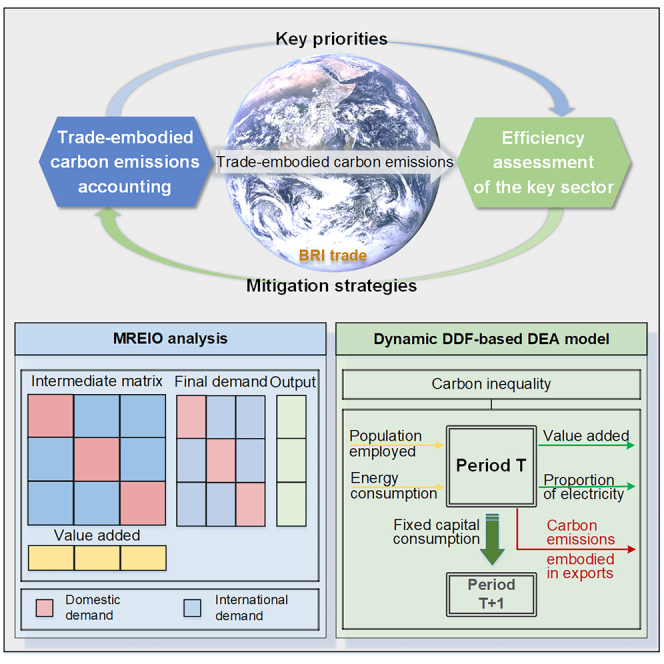

## Introduction

### Research background

The escalation of carbon emissions, as a principal driver of climate change, has threatened the stability of natural and human systems with broad and profound implications.^1^^,^^2^ This phenomenon not only exacerbates the greenhouse effect and leads to a warmer planet but also triggers a ripple effect of extreme weather events including heatwaves, droughts, melting ice caps, and biodiversity decrease.^3^ On the other side, trade globalization has resulted in a growing geographical disconnect between the environmental implications and consumption drivers, leading to considerable carbon emissions embodied in traded commodities.^4^^,^^5^^,^^6^^,^^7^ These negative environmental exogeneities counteract global mitigation efforts and may hinder the implementation of the Sustainable Development Goals (SDGs).^8^^,^^9^ Quantifying the potential impacts of trade-embodied carbon emissions is pivotal for climate change mitigation strategies.^8^ However, traditional practices merely account for the superficial distribution of carbon emissions, failing to reveal their fundamental origin sources.^10^ In this context, the integration of consumption-based environmental indicators to prevent any loopholes in sustainability assessments is imperative, as evidenced by a growing body of studies.^11^^,^^12^^,^^13^^,^^14^^,^^15^

The Belt and Road Initiative (BRI) exemplifies the complexities of modern trade networks, involving 151 countries and over 30 international organizations and profoundly influencing the global economic dynamics.^16^^,^^17^ So far, the BRI has covered 64.8% of the population and 40.9% of the gross domestic product (GDP) globally.^18^ Additionally, the aggregate trade volume between China and BRI countries surged to 2.1 trillion US dollars in 2022.^19^ As the BRI has evolved into a primary driver of global trade and investment,^20^^,^^21^ it has also resulted in considerable trade-embodied carbon emissions.^5^^,^^13^ This emphasizes the need for nuanced research on accounting and mitigating trade-embodied carbon emissions within such a global project.^22^ However, the accounting of trade-embodied carbon emissions among BRI countries has been lacking since 2016, let alone rigorous mitigation strategies. Additionally, the COVID-19 pandemic has magnified the complexities of globalization, trade exchanges, and environmental pollutants, complicating the trace and mitigation of trade-embodied carbon emissions.^23^ This crisis has underscored the frailties and sustainability of global trade networks, pressing the need for environmentally sustainable trade practices that can withstand not only health crises but also the broader impacts of climate change.^24^ Therefore, it is crucial to construct a flexible methodological framework to bridge the gap between accounting for trade-embodied carbon emissions and supporting optimal decision-making on deep mitigation.

### Literature review

Extensive studies have sought to link carbon emissions explicitly with global trade flows. However, traditional practices of carbon emissions accounting rely on the production allocation principle, ignoring the intricate network of indirect emissions.^13^^,^^25^^,^^26^ In this context, a consumption-based accounting approach is proposed to capture the carbon emissions triggered and consumed by final consumers,^11^^,^^27^ which can reveal a more holistic assessment of carbon emissions.^10^ Advances in multi-regional environmental input-output (MREIO) analysis open up the possibility of mapping out in-depth carbon emission transfers among multiple regions and international organizations.^13^^,^^28^^,^^29^

As a cornerstone method, MREIO analysis is widely applied in the accounting of carbon emissions on multiple levels (see Table 1).^13^^,^^14^^,^^29^ Simultaneously, some research associated carbon emission accounting with specific issues, such as carbon inequality, extreme poverty, resource security risks, and COVID-19 pandemic, revealing complex pathways of carbon emissions across global sustainable development.^30^^,^^31^^,^^32^^,^^33^ With the process of globalization and integration, substantial carbon emissions are embodied in international trade, named trade-embodied carbon emissions.^34^^,^^35^^,^^36^ This paradigm offers novel insights for precisely assessing the environmental and social impacts of globalization, and some scholars have conducted the quantification of trade-embodied carbon emissions.^5^^,^^12^^,^^20^^,^^28^^,^^32^^,^^37^ Moreover, MREIO analysis opens up the possibility of identifying key priorities (i.e., countries and sectors) for providing mitigation options. Specifically, there are four distinct ways including the magnitude of carbon emissions,^26^^,^^36^^,^^38^ carbon emission transfer pathways,^39^^,^^40^ main driving forces through power-of-pull approach,^39^^,^^41^ and the central sector of carbon flows identified by social-ecological network analysis.^42^ These scholarships significantly broaden the knowledge base and inform policies and regulations for carbon emission mitigation.

**Table 1:** Literature list on accounting of carbon emissions on a global scale

Author	Region	Country	Time frame	Allocation principle
Bruckner et al.^30^	Global countries	116	2011	Consumption-based carbon emissions
Chancel et al.^31^	Global countries	116	1990–2019	Consumption-based carbon emissions
Davis et al.^37^	Global countries	113	2004	Trade-embodied carbon emissions
Fang et al.^5^	BRI countries	65	2015	Trade-embodied carbon emissions
Han et al.^20^	BRI countries	65	2012	Trade-embodied carbon emissions
Liu et al.^32^	Global countries	14	2019	Trade-embodied carbon emissions
Lu et al.^13^	BRI countries	60	1995–2015	Production-based carbon emissions
Meng et al.^12^	Developing countries	129	2004–2011	Trade-embodied carbon emissions
Su and Ang^28^	Global countries	9	2000	Trade-embodied carbon emissions
Taherzadeh et al.^33^	Global countries	189	1990–2015	Consumption-based carbon emissions
Tian et al.^43^	BRICS countries	5	1995–2015	Environmental emissions
Tukker et al.^29^	EU countries	27	2007	Consumption-based carbon emissions
This study	BRI countries	66	2015–2019	Trade-embodied carbon emissions

Numerous studies have pointed out that enhancing efficiency is a critical path to carbon emission mitigation, and the first step is to assess it.^44^^,^^45^ Data envelopment analysis (DEA) obviates the necessity of preprocessing and offers a valuable way to assess the relative efficiency of a cluster of homogeneous decision-making units (DMUs) based on the distance function.^46^ The assessments of carbon emission efficiency based on DEA models within global countries have proliferated (see Table 2).^47^^,^^48^^,^^49^^,^^50^^,^^51^^,^^52^^,^^53^^,^^54^ Slacks-based measure and directional distance function (DDF) are two extensively adopted distance functions. Despite DEA models based on slacks-based measure offering a more accurate assessment of efficiency by considering the perspective of slack variables,^55^ the efficiency rankings may lack explanatory capability due to the influence of non-negative radial slack variables.^54^^,^^56^ DDF-based DEA models can fully tap the potential of improving efficiency by defining different direction vectors to designate the improvement direction of inputs/outputs.^57^^,^^58^ In addition, considering that a dynamic view enables to capture minor changes of efficiency within a continuous process across periods,^56^ this study constructs a dynamic DDF-based DEA model to assess carbon emission efficiency.

**Table 2:** Literature list on assessments of carbon emission efficiency on a global scale

Reference	Model	Region	Level	Number	Time frame	Temporal view
Feng et al.^47^	DDF	Global countries	Country	165	2000–2014	Static
Iram et al.^48^	Malmquist index analysis	OCED countries	Country	26	2013–2017	Dynamic
Kortelainen^49^	Malmquist index analysis	European countries	Country	20	1990–2003	Dynamic
Wang et al.^50^	SBM	APEC countries	Country	16	2001–2010	Static
Woo et al.^51^	Malmquist index analysis	OCED countries	Country	31	2004–2011	Dynamic
Xie et al.^52^	Malmquist index analysis	OCED and BRIC countries	Electric power	30	1996–2010	Dynamic
Zhang et al.^53^	Window analysis	European countries	Country	9	2010–2014	Dynamic
Zhou et al.^54^	SBM	OCED countries	Country	26	1998–2002	Dynamic
This study	DDF	BRI	Manufacturing	63	2015–2019	Dynamic

### Research hypotheses

The BRI has played a pivotal role in fostering economic integration and facilitating transnational production through the supply chain network. Within this intricate network, the assumption of constant returns to scale helps to simplify the efficiency assessment in sectoral production activities, ensuring that efficiency scores remain unbiased toward any scale of production.^59^ Moreover, the BRI has significantly promoted the exchange and collaboration of technologies, which has led to a convergence in production techniques.^60^^,^^61^ Through regional cooperative mechanisms, BRI countries shared similar policy frameworks and development environments.^16^ Thus, several fundamental assumptions are applied to the efficiency evaluation of the identified key sectors in the BRI, including constant returns to scale, technological homogeneity, and similar external environmental factors.

Efficiency plays an indispensable link in achieving carbon neutrality. Enhancing the efficiency of energy production and utilization promotes carbon emission reduction without hindering economic growth.^48^ Numerous developed countries have managed to decouple economic growth from environmental emissions through technological innovation and efficiency enhancement.^62^ This fact signals the feasibility of transitioning to a low-carbon economy driven by clean energy and sophisticated regulatory frameworks, which are far from reach for emerging countries. Carbon inequality, characterized by the disproportionate distribution of global carbon emissions among the population,^31^ stands as a huge challenge in global carbon emission mitigation.^63^ With the increase of carbon inequality, low-income groups often lack the resources necessary to shift toward clean energy and technologies, relying on cheap but high-carbon alternatives instead. On the other hand, the consumption activities of high-income groups are commonly carbon intensive, not only because they can afford higher energy costs but also due to their extensive and luxurious lifestyle.^64^ Based on the earlier analysis, we propose the following hypotheses: (1) enhancing efficiency of the identified key sector can mitigate trade-embodied carbon emissions and (2) carbon inequality plays a negative moderating role in the relationship between carbon emission efficiency and carbon emissions.

### Motivation and contribution

Surveying prior research, the intricate nexus between international trade and carbon emissions has been extensively explored.^4^^,^^5^^,^^6^^,^^7^ Recent studies have conducted systematic assessments of trade-embodied carbon emissions under a global scope using MREIO analysis. However, these studies lack the latest accounting results, especially for BRI countries, thereby falling short in guiding carbon emission mitigation strategies. Therefore, accounting for trade-embodied carbon emissions and identification of the key priorities among BRI countries need to be further investigated for more information.^4^ Concurrently, efficiency enhancement has been acknowledged as a pivotal route for carbon emission mitigation.^44^^,^^45^^,^^48^ Numerous scholars have conducted the assessments of carbon emission efficiency at national and sectoral scales, with DEA playing a crucial tool in efficiency assessment.^47^^,^^48^^,^^65^^,^^66^ While the profound impact of carbon inequality on carbon emission efficiency is well accepted,^63^^,^^64^ the dynamic characteristics of carbon emission efficiency considering the impact of carbon inequality in BRI countries at sectoral scale have not been thoroughly investigated. Overall, previous studies only partially meet the requirements for carbon emission mitigation in the BRI, highlighting the urgency for a comprehensive research framework to address this issue.

To fill these knowledge gaps, this study attempts to account for trade-embodied carbon emissions among BRI countries and goes a step further in the mitigation path by improving the carbon emission efficiency of the identified key priorities. Initially, MREIO analysis is adopted to account for trade-embodied carbon emissions within BRI countries from 2015 to 2019, and then the outcomes are utilized to identify the key priorities. Subsequently, a dynamic DDF-based DEA model in the presence of a non-discretionary variable is constructed to assess the carbon emission efficiency of the identified key sector in BRI countries and to seek the improvement path.

The marginal contributions of this study can be summarized as follows: (1) accounting for trade-embodied carbon emissions within BRI countries from 2015 to 2019, (2) identifying the key priorities of trade-embodied carbon emissions within BRI countries, (3) constructing a dynamic DDF-based DEA model for assessing carbon emission efficiency of the identified key sector, and (4) proposing cooperation and common development interventions to mitigate trade-embodied carbon emissions among BRI countries deeply.

## Results and discussion

### Total trade-embodied carbon emissions

**Figure 1:**
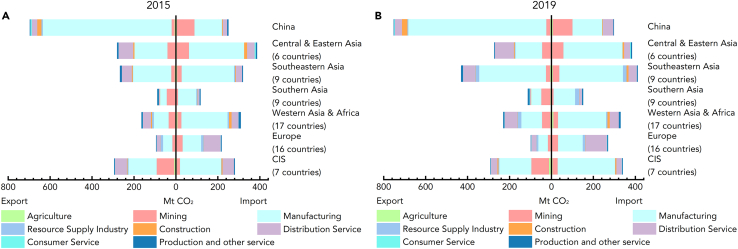
Spatial distribution and sectoral composition of trade-embodied carbon emissions in BRI countries

Initially, we account for the trade-embodied carbon emissions within BRI countries, and the detailed spatial distribution and sectoral composition are illustrated in Figure 1. At the macro scale of the BRI, trade-embodied carbon emissions increased by 16.03%, rising from 1883.54 million metric tons (Mt) in 2015 to 2185.41 Mt in 2019. On the regional scale, despite overall consistent trends, significant disparities existed among BRI regions with dynamic shifts between 2015 and 2019. In terms of emissions embodied in exports, China maintained the foremost position from 2015 (696.25 Mt) to 2019 (752.06 Mt). The region with the smallest emissions shifted from Southern Asia in 2015 (88.56 Mt) to Europe in 2019 (98.12 Mt). In terms of emissions embodied in imports, the region with the largest emissions also shifted from Central and Eastern Asia (excluding China) in 2015 (387.18 Mt) to Southeastern Asia in 2019 (411.34 Mt). Southern Asia, on the other hand, ranked lowest in both 2015 (117.71 Mt) and 2019 (151.23 Mt).

When it comes to the carbon emission characteristics (i.e., importers or exporters) within BRI countries, existing literature demonstrated that the BRI as a whole acted as a net exporter.^13^^,^^20^^,^^67^ Furthermore, our findings indicated that most BRI regions were net importers, such as Central and Eastern Asia (excluding China), Southern Asia, Western Asia and Africa, and Europe. This discrepancy primarily results from China, an absolute net exporter, with exports tripling its imports in 2015 and reducing slightly to 2.5 times in 2019 (see Figure 1). This further informs the importance of coordinating climate governance and accelerating carbon neutrality efforts in BRI regions. On the other hand, the results present concrete evidence of role transitions between net importers and exporters within BRI regions. For instance, the Commonwealth of Independent States (CIS) transitioned from a net exporter in 2015 to a net importer in 2019, while Southeastern Asia underwent the opposite transition. This indicates the opportunity to reverse the carbon emission roles of BRI regions through a series of mitigation strategies.

Regarding the sectoral composition of trade-embodied carbon emissions, BRI regions exhibited similar structures. Whether for emissions embodied in imports or exports, a substantial proportion of trade-embodied carbon emissions originated from the manufacturing sector in each region. This is mainly because the manufacturing sector stands as the main contributor to global carbon emissions due to its energy-intensive nature.^68^ Hence, addressing carbon emission mitigation within this sector has become critically imperative.

### Trade-embodied carbon flows

**Figure 2:**
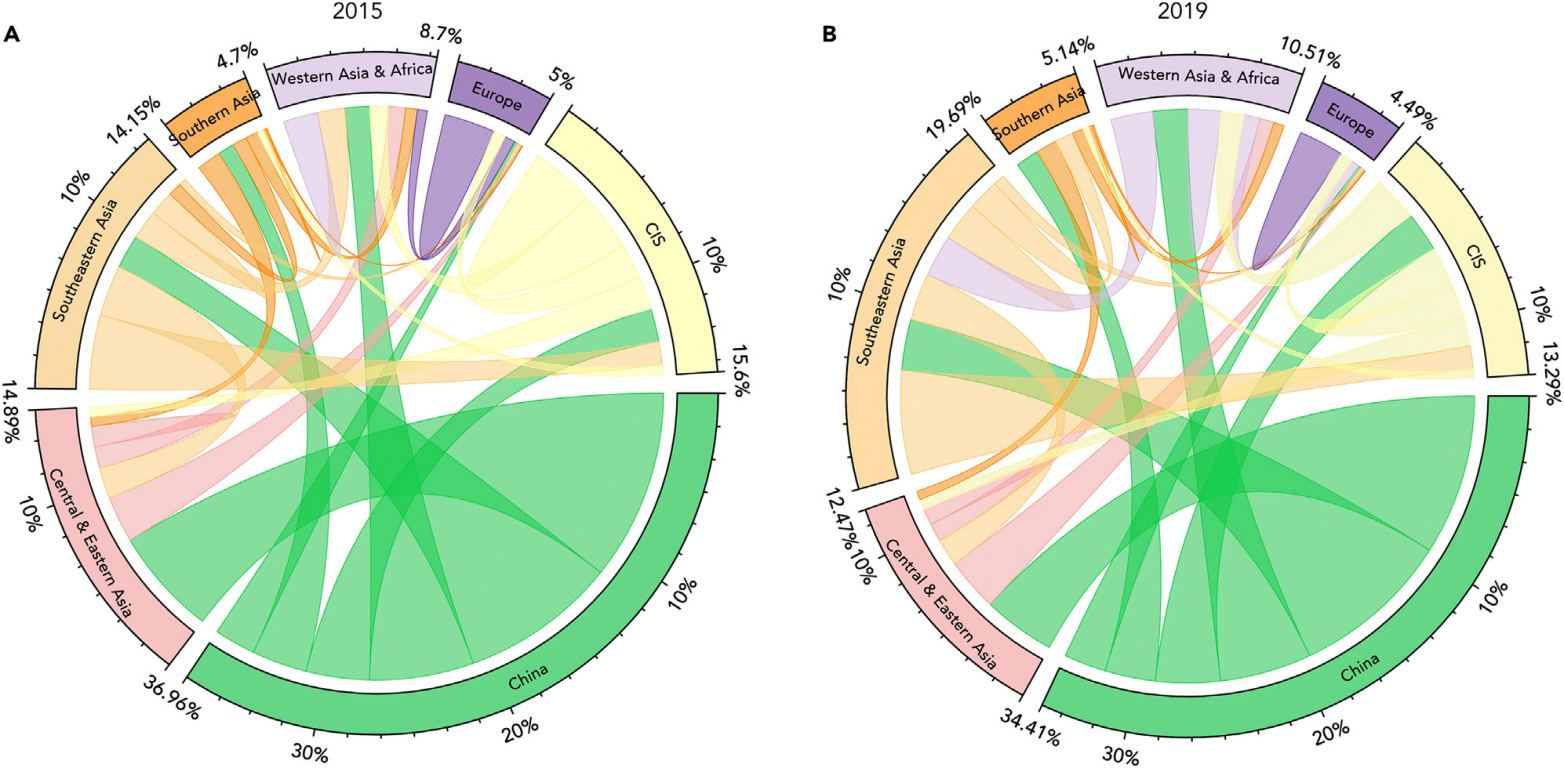
Trade-embodied carbon flows among BRI regions

Given the intricate and globalized network of trade-embodied carbon flows between countries and sectors, we map out the primary carbon flows between and within the BRI on multiple scales. On the region scale, we observe both inter-regional and extra-regional dynamic transfers between 2015 and 2019 (see Figure 2). Notably, despite China’s emissions embodied in exports increased overall (see Figure 1), the proportion decreased by 6.90% from 2015 (36.96%) to 2019 (34.41%). In addition, Central and Eastern Asia (excluding China) showed a slight decrease. Conversely, the proportion of Southeastern Asia increased by 39.15% from 2015 (14.15%) to 2019 (19.69%), demonstrating a trend of rapid growth. The expansion of trade-embodied carbon emissions in Southeastern Asia mirrors the negative environmental impact of its rapid economic growth and loose environmental regulations.^12^^,^^13^

**Figure 3:**
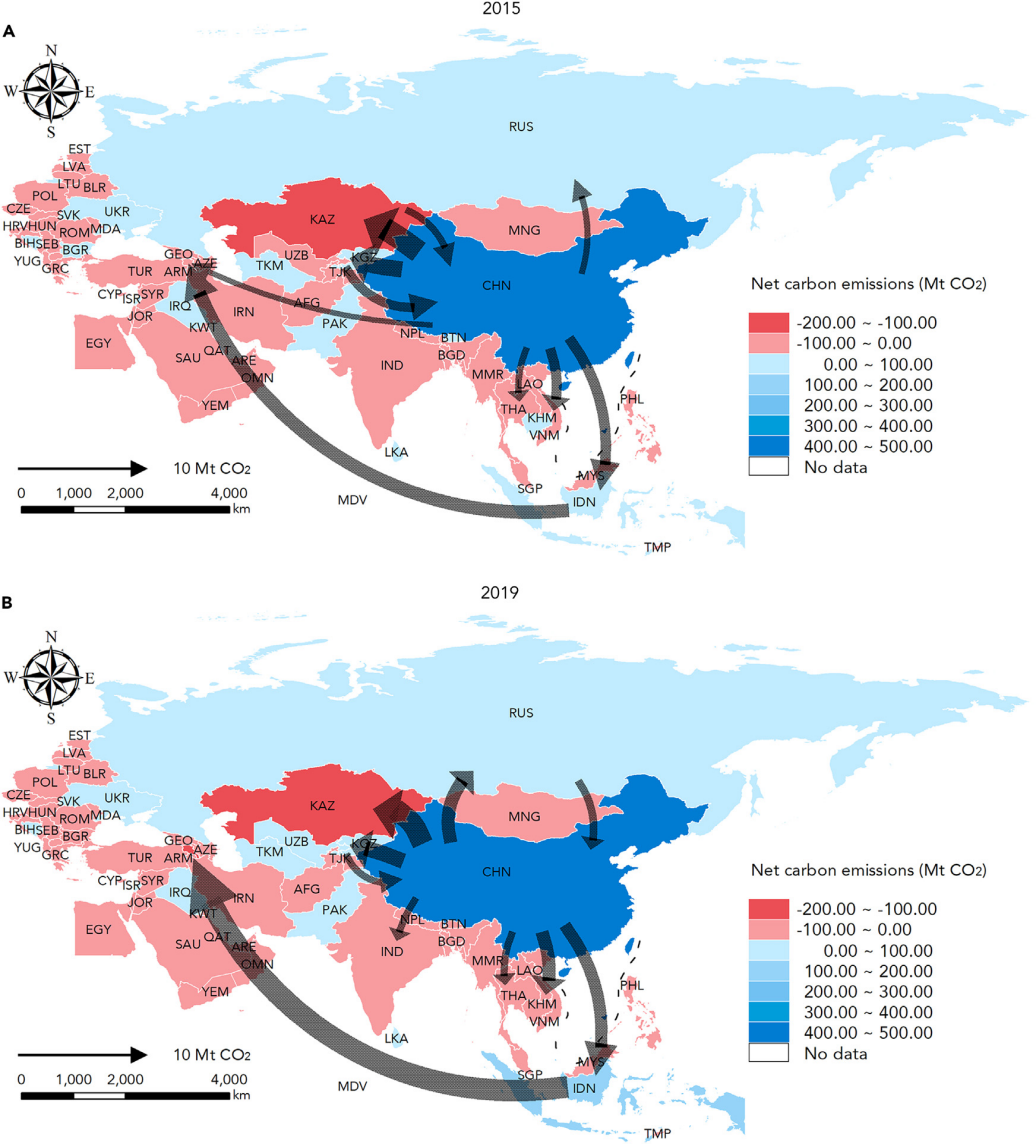
Net trade-embodied carbon flows in BRI countries

On the country scale, the top ten net trade-embodied carbon flows between 2015 and 2019 are illustrated in Figure 3, with blue representing the net exporters and red representing the net importers. China stood as an absolute net exporter, accounting for more than one-third of the total trade-embodied carbon emissions in the BRI (see Figure 2). On the contrary, Kazakhstan was identified as the largest net importer due to its substantial exports of raw materials and energy resources. This contrast is largely attributed to the trade-embodied carbon flows transferred from China to Kazakhstan, with an annual carbon emission volume exceeding 110 Mt. As an adjacent and intimate trading partner of China, Kazakhstan has maintained a resilient trade relationship with China since its independence,^19^ yet widespread industrial exports have exacerbated the imbalanced carbon transfer.^69^ Regarding the time dimension, the major carbon transfer pattern in the BRI remained unchanged, with most of the trade-embodied carbon flows associated with China. The extensive and well-established supply chain network, coupled with logistical advantages of maritime transportation, positioned China as a pivotal global hub.^4^^,^^12^^,^^16^ Remarkably, the colors of Southeastern Asia countries, especially Indonesia, were deepened from 2015 to 2019. This indicates the rise of emerging economies due to their growing economic influence and industrialization efforts. As the largest economy in Southeastern Asia, Indonesia has become increasingly attractive to manufacturing firms with its low-cost labor market and evolving industrialization.^70^

### The key priorities in BRI countries

While the landscape of environmental impacts of international trade in BRI countries is a cause for concern, several key countries/sectors can be viewed as the entry point to mitigate climate change. To identify the key countries for carbon emission mitigation, we further account for the trade-embodied carbon emissions of each country, and the top ten countries are exhibited in Tables 3 and 4. In terms of emissions embodied in imports, the top ten countries’ carbon emissions ranged from 60 Mt to 300 Mt, demonstrating a relatively even distribution. The top ten countries remained the same over the first four years, only with changes in the rankings. In 2019, Syria replaced the United Arab Emirates as the tenth largest importer. Emissions embodied in exports showed a starkly different picture, with emissions ranging from 40 Mt to 760 Mt and displaying significant disparities. Notably, China and Indonesia held the first and second positions from 2015 to 2019, respectively. Following these two countries, Russia and Kyrgyzstan sequentially placed in the rankings, with fluctuations in their positions between 2015 and 2019.

**Table 3:** Top ten countries of emissions embodied in imports in BRI countries

2015		2016		2017		2018		2019	
Country	Import	Country	Import	Country	Import	Country	Import	Country	Import
CHN	250.75	CHN	286.07	KAZ	243.02	CHN	281.08	CHN	297.98
KAZ	230.29	KAZ	210.27	CHN	234.95	KAZ	216.42	KAZ	211.94
KGZ	140.87	ARM	141.08	KGZ	150.55	ARM	144.36	ARM	187.11
ARM	136.21	KGZ	134.07	ARM	147.66	KGZ	140.23	KGZ	148.09
IDN	111.87	IDN	114.89	RUS	127.30	RUS	128.10	IDN	139.18
RUS	102.76	RUS	105.76	IDN	118.98	IDN	126.25	VNM	105.69
ARE	74.49	VNM	72.85	VNM	81.78	VNM	87.27	RUS	90.01
VNM	73.13	THA	72.14	ARE	76.35	THA	78.39	THA	89.74
THA	67.82	CZE	67.04	THA	73.19	ARE	70.86	CZE	67.92
CZE	66.88	ARE	62.18	CZE	69.93	CZE	68.83	SYR	65.42

**Table 4:** Top ten countries of emissions embodied in exports in BRI countries

2015		2016		2017		2018		2019	
Country	Export	Country	Export	Country	Export	Country	Export	Country	Export
CHN	696.25	CHN	663.07	CHN	681.62	CHN	679.29	CHN	752.06
IDN	149.85	IDN	153.90	IDN	169.50	IDN	194.11	IDN	277.26
KGZ	146.97	KGZ	148.52	RUS	166.18	RUS	185.88	RUS	180.99
RUS	134.46	RUS	124.48	KGZ	146.91	KGZ	162.73	KGZ	150.74
KAZ	92.65	KAZ	84.14	ARM	85.33	KAZ	87.55	VNM	84.10
ARM	77.17	SYR	81.30	KAZ	85.17	ARM	82.03	KAZ	75.27
UKR	63.31	ARM	77.35	SYR	75.59	THA	62.05	SYR	61.59
THA	57.61	THA	63.61	THA	64.83	VNM	61.39	IND	59.42
VNM	42.74	UKR	56.23	VNM	45.37	IND	52.92	ARM	45.13
SYR	40.40	VNM	42.59	UKR	43.36	IRQ	49.83	UKR	42.14

The intersection of the top ten countries in emissions embodied in imports and exports reveals a core group of countries, including China, Indonesia, Kazakhstan, Kyrgyzstan, Armenia, Russia, Vietnam, Thailand, and Syria. China, as the largest manufacturing factory, primarily derives high carbon emissions from its vast industrial sector and energy-intensive industries.^71^ Russia and Kazakhstan are both abundant in energy resources, and the production process of energy products such as oil and natural gas consists of a series of mining and energy-consumption activities, resulting in substantial carbon emissions.^72^ Indonesia, Vietnam, and Thailand are three fast-growing emerging economies, with significant progress in manufacturing, and, in turn, this has also contributed to an increase in carbon emissions.^70^ Kyrgyzstan, Armenia, and Syria are landlocked countries heavily reliant on agriculture and mining sectors, and the main contributors to their carbon emissions are energy consumption and transportation. Additionally, the prolonged internal conflict and war in Syria have resulted in economic devastation, constraining its capacity to manage environmental regulations and reduce carbon emissions. Based on the wide-ranging ripple effects on the total amount of trade-embodied carbon emissions around BRI countries, these countries are identified as key countries.

**Figure 4:**
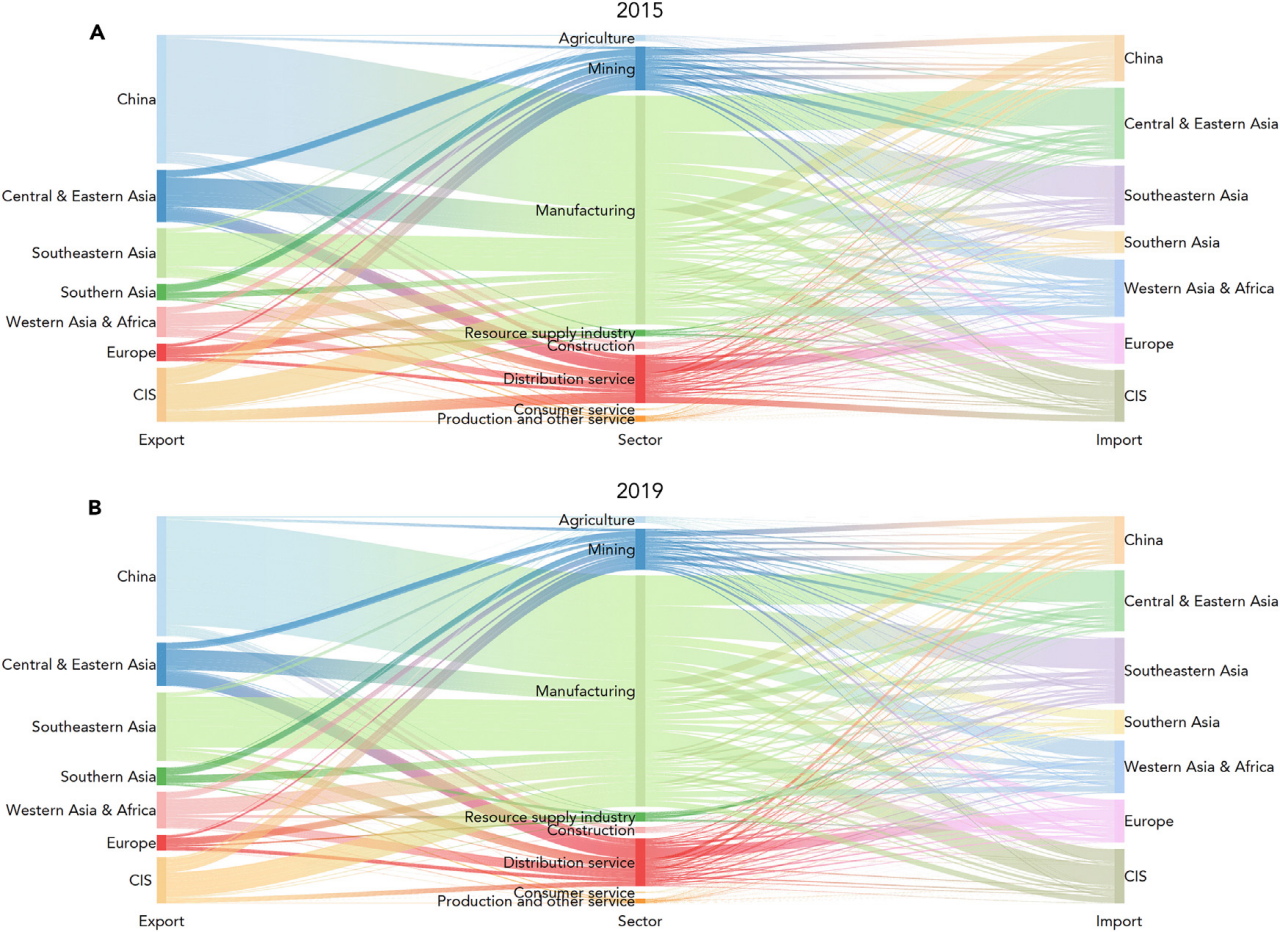
Inter-regional and inter-sectoral linkage of trade-embodied carbon emissions in the BRI in 2015 and 2019

In focusing on the key sector, the manufacturing sector emerges as the primary source of trade-embodied carbon emissions. It can be observed that emissions embodied in the manufacturing sector outweighed the sum of trade-embodied carbon emissions in other sectors from 2015 to 2019 annually (see Table 5). Simultaneously, emissions embodied in this sector sustained a rapid growth rate, increasing by 17.4% from 2015 (1235.95 Mt) to 2019 (1450.92 Mt). In terms of composition, China accounted for approximately 50% of emissions in the manufacturing sector, making it the largest contributor (see Figure 4). Collectively, Southeastern Asia, Central and Eastern Asia (excluding China), Western Asia and Africa, and CIS were the top four importers of emissions embodied in the manufacturing sector, accounting for more than 15% proportion individually and 75% proportion taken together. This implies that the enhancement of domestic product quality management in emerging economies has gradually attracted manufacturing enterprises to enter their markets, taking over China’s market share.^73^

**Table 5:** Trade-embodied carbon emissions of each sector in the BRI from 2015 to 2019

Sector	2015	2016	2017	2018	2019
Manufacturing	1235.95	1272.95	1299.77	1309.09	1450.92
Distribution service	260.28	275.99	333.12	352.91	299.80
Mining	236.02	199.26	212.56	257.97	259.27
Construction	40.76	34.31	35.53	39.75	38.06
Agriculture	34.14	37.21	38.40	39.93	42.62
Resource supply industry	33.94	37.26	37.47	40.49	57.83
Production and other service	33.37	34.42	35.06	35.90	28.90
Consumer service	9.08	9.08	7.69	7.36	8.01

The manufacturing sector plays a vital role in the dynamics of trade globalization.^12^ The rapid expansion of the manufacturing sector has contributed to global economic prosperity, but it has also caused an increase in carbon emissions due to its substantial energy consumption simultaneously. This phenomenon is particularly pronounced in developing countries, largely due to the effect of pollution havens.^74^ This paradox underscores the complex balance between industrial expansion and environmental sustainability. Consequently, this study identifies the manufacturing sector as the key sector for carbon emission mitigation in the BRI.

### Trade-embodied carbon emission efficiency

The overall trade-embodied carbon emission efficiency of the manufacturing sector in BRI countries stood at 0.6268 on average, with an inefficiency of 0.3732, indicating considerable room for enhancement. Given the highly diverse socioeconomic conditions across the countries, we illustrate the overall and annual efficiency in Table 6. Out of the 63 countries evaluated, 15 countries achieved an optimal efficiency of 1, such as China, Poland, Singapore, Turkey, etc. In 2019, the number of countries reaching the frontier increased to 18. Notably, most key countries obtained lower efficiency scores, with China and Indonesia standing as exceptions. These results witness the shift toward a higher position in the global value chain through thriving developments and technological innovation of the manufacturing sector in both China and Indonesia.^75^ Furthermore, China diligently tackles environmental challenges through a rigorous regulatory framework that drives manufacturing enterprises to cleaner and more efficient operations. Conversely, Russia’s manufacturing sector exhibited merely the average level of trade-embodied carbon emission efficiency. This reflects the uncertainty of whether the implementation of environmentally conscious policies can be effectively carried out under current global environmental standards, even with advanced technological capabilities.^76^

**Table 6:** Trade-embodied carbon emission efficiency of the manufacturing sector in 2015 and 2019

Country	Overall	2015	2019	Country	Overall	2015	2019	Country	Overall	2015	2019
POL	1.00	1.00	1.00	ROU	0.71	0.51	0.79	SER	0.44	0.37	0.58
BRN	1.00	1.00	1.00	JOR	0.67	0.46	0.83	HUN	0.43	0.44	0.42
KHM	1.00	1.00	1.00	SVK	0.67	0.50	0.77	HRV	0.43	0.42	0.44
CHN	1.00	1.00	1.00	BHR	0.65	0.66	0.62	THA	0.42	0.44	0.43
IRN	1.00	1.00	1.00	EST	0.65	0.65	0.70	EGY	0.42	0.41	0.42
MDV	1.00	1.00	1.00	BTN	0.65	0.77	0.62	BGR	0.41	0.41	0.42
MNE	1.00	1.00	1.00	CYP	0.64	0.59	0.64	UZB	0.41	0.35	0.36
PHL	1.00	1.00	1.00	RUS	0.61	0.61	0.62	KAZ	0.37	0.40	0.34
QAT	1.00	1.00	1.00	MYS	0.58	0.55	0.62	BLR	0.37	0.37	0.39
SGP	1.00	1.00	1.00	LTU	0.58	0.49	0.69	ARM	0.36	0.35	0.35
TJK	1.00	1.00	1.00	MKD	0.58	0.42	0.75	UKR	0.35	0.34	0.37
TUR	1.00	1.00	1.00	SVN	0.58	0.48	0.67	LAO	0.35	0.33	0.35
CZE	1.00	1.00	1.00	LBN	0.58	0.43	0.55	NPL	0.33	0.25	0.30
ISR	1.00	1.00	1.00	IND	0.57	0.57	0.57	KGZ	0.32	0.30	0.33
SAU	1.00	1.00	1.00	VNM	0.54	1.00	0.33	AFG	0.32	0.26	0.28
BGD	0.88	0.50	1.00	KWT	0.54	0.41	0.45	IRQ	0.32	0.30	0.22
BIH	0.87	0.58	1.00	AZE	0.53	0.42	0.69	LKA	0.31	0.24	0.29
MNG	0.83	0.80	0.89	PAK	0.51	0.51	0.51	OMN	0.31	0.45	0.12
IDN	0.79	1.00	0.65	GEO	0.50	0.41	0.44	MMR	0.30	0.21	0.30
GRC	0.77	0.54	1.00	LVA	0.49	0.41	0.41	ARE	0.18	0.11	0.17
ALB	0.75	0.76	0.62	MDA	0.45	0.36	0.41	YEM	0.13	0.27	0.03

**Figure 5:**
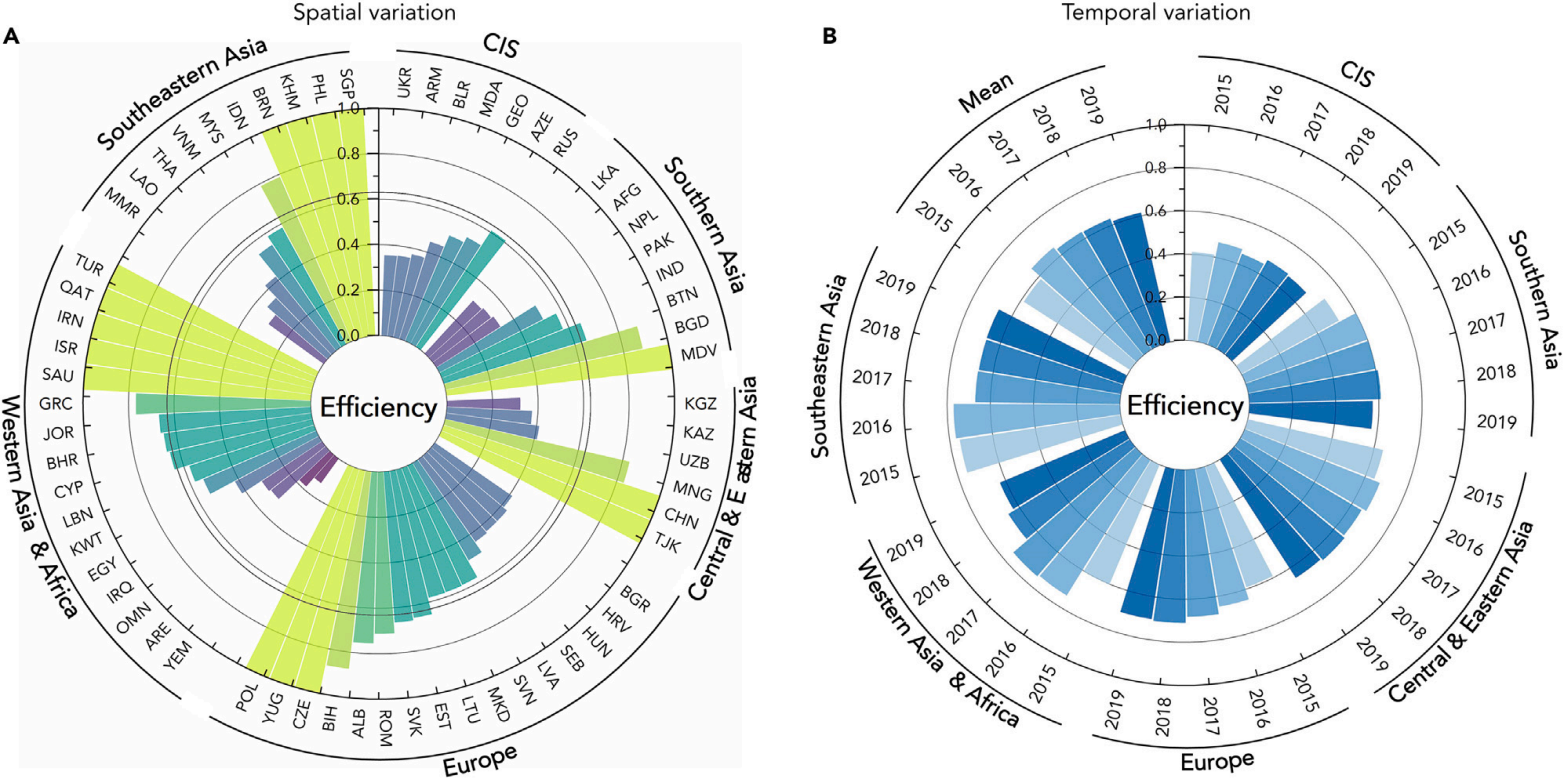
Spatial and temporal variation of trade-embodied carbon emission efficiency of the manufacturing sector

Based on the results of the efficiency evaluation, a significant imbalance in the efficiency across and within BRI regions was observed (see Figure 5A). Unlike other regions, where at least one country reached the production frontier with an efficiency of 1, none of the CIS countries achieved it. Among the regions, CIS was evaluated as the least efficient region, with an efficiency of 0.4529 on average. Compared to other developed countries and regions, the adoption of clean energy in CIS progresses at a slow pace, leading to lower efficiency.^77^ On the contrary, Southeastern Asia exhibited the optimal level among the regions, followed by Europe, Western Asia, and Africa, with efficiency of 0.6980, 0.6629, and 0.6563 on average, respectively. This can be explained by the gradually formed manufacturing industry cluster in Southeastern Asian countries, coupled with policy support and low labor costs.^70^ Furthermore, there were also significant efficiency gaps within each distant region, with the gap between the lowest and highest exceeding 2-fold. The discrepancy was most pronounced in Western Asia and Africa, where Yemen’s efficiency was nearly 7-fold lower than the highest. Regarding temporal dynamics, the annual efficiency in BRI regions fluctuated from 2015 to 2019 but exhibited a slight growth over the five years (see Figure 5B). The dynamic characteristics of the annual efficiency in BRI regions can be divided into two major categories. Some regions (i.e., Europe, Western Asia and Africa, and Southeastern Asia) experienced a single peak with different turning points. Other regions (i.e., Central and Eastern Asia, CIS, and Southern Asia) witnessed two significant peaks in 2016 and 2018. Most regions reached their maximum values in 2018, followed by a downturn, while Central and Eastern Asia reached their maximum value in 2016. The average efficiency in the BRI had a single peak in 2016 affected by these compound factors.

**Figure 6:**
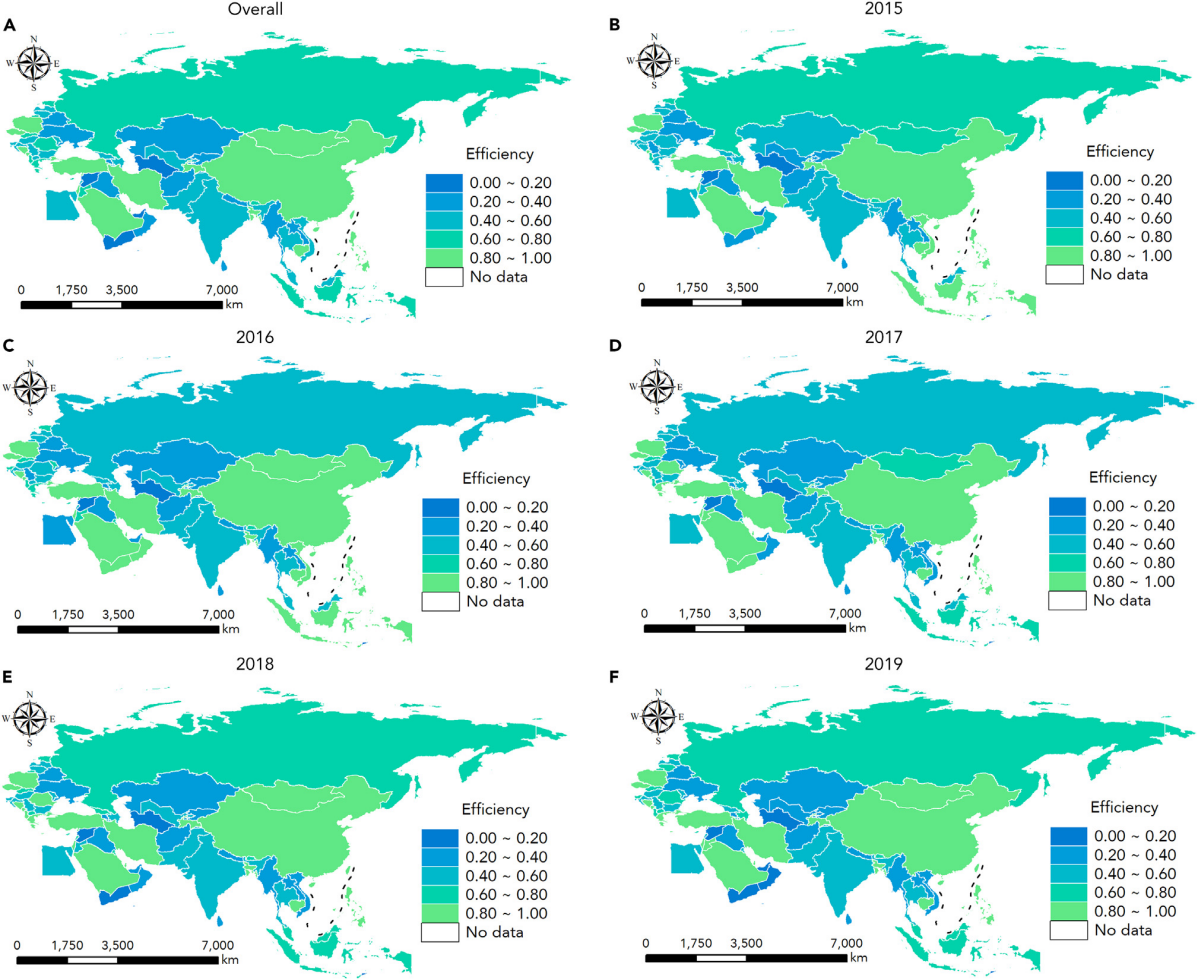
Geographical distribution of trade-embodied carbon emission efficiency of the manufacturing sector from 2015 to 2019

A more detailed geographical distribution of trade-embodied carbon emission efficiency of the manufacturing sector in BRI countries from 2015 to 2019 is illustrated in Figure 6. There appeared to be an underlying connection between the trade-embodied carbon efficiency and net trade-embodied carbon emissions. Except for China, Indonesia, Russia, and Kazakhstan, the remaining regions exhibited higher efficiency as net importers and lower efficiency as net exporters (see Figures 3 and 6). Despite being a net importer, Kazakhstan exhibited a relatively low efficiency of 0.3739. This may be associated with its considerable emissions embodied in exports (see Table 4). Notably, whilet China had substantial trade-embodied carbon emissions, its efficiency consistently maintained a leading position. This can be attributed to superlative efforts in carbon emission mitigation, which have compelled Chinese manufacturing firms to enhance their resource utilization efficiency.^78^

### Patterns between carbon emissions and carbon emissions efficiency

**Figure 7:**
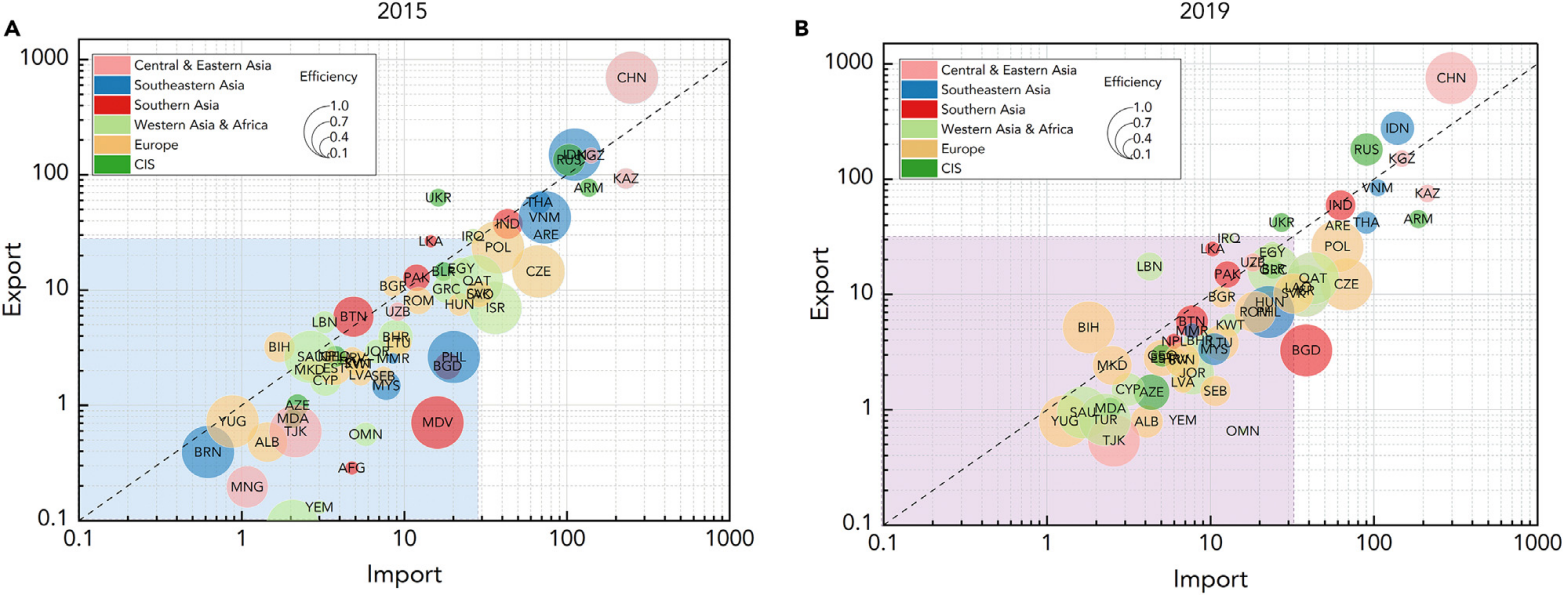
The patterns of trade-embodied carbon emissions and efficiency of the manufacturing sector The logarithmic transformation has been applied to both axes to facilitate analysis. The diagonal line represents the benchmark that emissions embodied in imports equals exports, with bubbles above the line indicating net exporters and bubbles below indicating net importers.

This study reveals potential development patterns in the dynamics of trade-embodied carbon emissions and efficiency of the manufacturing sector in Figure 7. Similar to the regional role transitions, these shifts also occurred within BRI countries. Bhutan, Bulgaria, and Cambodia shifted from net exporters to net importers, while Uzbekistan shifted from a net importer to a net exporter. Additionally, it can also be noted that the distribution of emissions embodied in imports and exports in the BRI changed from scattered to aggregated. This indicates that economic interconnection and intercommunication among BRI countries have been strengthened with the implementation and advancement of the initiative.^79^ Simultaneously, the initiative has propelled industrial transformation and upgrading, resulting in the transition of countries along the route from dispersion to aggregation.^16^ This shift is advantageous for the economic concentration and coordinated development of the region, thereby enhancing carbon emission efficiency.^80^

Coupled with the efficiency of the manufacturing sector in each country, there was a notable improvement within the BRI from 2015 to 2019. This uptick was particularly significant among countries that initially reported lower efficiency in 2015. Moreover, the patterns of trade-embodied carbon emissions and efficiency of the manufacturing sector were observed: countries located above the diagonal line become more efficient when approaching the line, while those below the diagonal line become more efficient when moving away from the line.

To validate the correctness of our straightforward factual analysis, we further conduct an empirical analysis to examine the deep linkage between efficiency of the manufacturing sector, trade-embodied carbon emissions, and carbon inequality. We incorporate six variables in this section, categorized into dependent, independent, and control variables. The dependent variable is the sum of carbon emissions embodied in imports and exports, while the independent variable is trade-embodied carbon emission efficiency (CEE). Additionally, economic growth (GDP), industrial structure (Ind), health level (Hlt), and medical service level (Med) are selected as control variables to ensure the reliability of the empirical results. reliability of the results. First, we examine the impact of efficiency on trade-embodied carbon emissions by a baseline regression. Then, we introduce the interaction term between carbon inequality and efficiency to investigate the moderating effect of carbon inequality. The results of the baseline regression model and moderating effect model are reported in Table 7.

**Table 7:** The results of the baseline regression model and moderating effect model

Variable	Baseline regression model				Moderating effect model			
	Model (1)	Model (2)	Model (3)	Model (4)	Model (5)	Model (6)	Model (7)	Model (8)
	BRI	BRI	Exporter	Importer	BRI	BRI	Exporter	Importer
CEE	-0.091∗∗	-0.091∗	0.114	-0.122∗∗	0.230∗∗	0.240∗∗∗	0.679	0.180∗∗∗
	(-2.27)	(-1.89)	(0.73)	(-2.63)	(2.45)	(3.04)	(1.41)	(2.80)
CEE×Ceq					-0.013∗∗∗	-0.013∗∗∗	-0.026	-0.012∗∗∗
					(-4.42)	(-4.71)	(-1.07)	(-4.37)
GDP		-0.341∗	-0.669∗∗∗	-0.184		-0.360∗∗	-0.711∗∗∗	-0.196
		(-1.98)	(-3.96)	(-0.90)		(-2.04)	(-4.37)	(-0.94)
Ind		-0.446∗∗	-0.193	-0.428∗∗		-0.446∗∗	-0.288	-0.441∗∗
		(-2.08)	(-0.50)	(-2.22)		(-2.12)	(-0.70)	(-2.43)
Hlt		5.722	-2.716	7.122		5.207	-2.080	6.326
		(1.36)	(-0.46)	(1.410)		(1.27)	(-0.35)	(1.28)
Med		-0.024	0.023	0.163		-0.009	0.048	0.172
		(-0.22)	(0.13)	(1.35)		(-0.09)	(0.26)	(1.50)
Cons	2.902∗∗∗	-11.637	33.308	-22.964	2.940∗∗∗	-8.987	31.913	-19.189
	(130.13)	(-0.64)	(1.31)	(-1.13)	(122.19)	(-0.51)	(1.24)	(-0.97)
R-squared	0.991	0.991	0.997	0.990	0.991	0.992	0.997	0.991
Observations	315	315	64	244	315	315	64	244
Number of ID	63	63	13	50	63	63	13	50
Country FE	YES	YES	YES	YES	YES	YES	YES	YES
Year FE	YES	YES	YES	YES	YES	YES	YES	YES

In terms of the baseline regression results, the coefficients of CEE were significantly negative at the 5% and 10% confidence levels before and after incorporating control variables, respectively. It is evident that improving the efficiency of the manufacturing sector significantly mitigated trade-embodied carbon emissions, which is by the consensus that enhancing efficiency is an effective strategy for carbon emission mitigation.^44^^,^^45^^,^^48^ Furthermore, it convincingly demonstrates the effectiveness of mitigating carbon emissions by focusing on the key sector. In terms of the moderating effect model, the coefficients of CEE×Ceq were significantly negative at the 5% and 1% confidence levels before and after incorporating control variables, respectively. Existing literature has acknowledged the intricate impacts of inequality distribution of income or wealth on energy consumption and efficiency.^81^^,^^82^ This result implies carbon inequality has an inhibitory impact on the relationship between efficiency and trade-embodied carbon emissions, which expands the knowledge base in this field. At this point, both hypotheses in this study have been proved.

The aforementioned results may mask heterogeneity across countries, so this study further divides BRI countries into exporters and importers of trade-embodied carbon emissions for heterogeneity analysis. The carbon emission mitigation effect of efficiency and the negative moderating effect of carbon inequality were both significant at the 5% and 1% confidence levels for importers, respectively, while neither effect held statistical significance for exporters. This result reveals the presence of national heterogeneity in these two effects, corroborating the conclusions drawn by the academic community.^83^

Additionally, the coefficients of GDP and Ind were significantly negative across multiple groups, indicating that economic growth and industrial development can mitigate carbon emissions indeed. Furthermore, this suggests that some BRI countries have managed to decouple economic development from environmental pollution, which reveals the possibility of a win-win scenario for the economy and environment in the BRI.

### Conclusions

In this study, we adopt an MREIO analysis to account for trade-embodied carbon emissions and explore the inter-sectoral linkage in 66 BRI countries from 2015 to 2019. Subsequently, a dynamic DDF-based DEA model is constructed to assess the carbon emission efficiency of the identified key sector. Moreover, we conduct an empirical analysis to validate the impact of efficiency on carbon emissions and explore the moderating role of carbon inequality. The main conclusions can be summarized as follows.

(1) Trade-embodied carbon emissions in the BRI witnessed a steady increase during 2015–2019, primarily driven by the rise of Southeastern Asian countries. China, as the pivotal global hub in the BRI, contributed over one-third of carbon emissions. Regarding the sectoral distribution, the manufacturing sector was identified as the key sector in this study due to its substantial carbon emissions as the predominant contributor.
(2) In contrast to the substantial carbon emissions in the BRI, the overall efficiency of the manufacturing sector was 0.63 on average, with significant efficiency disparities both between and within BRI regions, indicating huge potential for enhancement. Nonetheless, the slow growth of efficiency signaled a positive trend, suggesting that BRI countries have made collective endeavors to mitigate climate change through industry aggregation.
(3) The positive role of efficiency on carbon emission mitigation was proved again, especially evident in net importers. Conversely, carbon inequality stood as a negative moderating role in this process. This underscores the effectiveness of targeting key sectors to mitigate global carbon emissions, with necessary vigilance to the rapid expansion of global inequality.

### Optimal carbon emission mitigation strategies

Du et al. in 2022^84^ noted that several BRI countries are located in climatically and geologically sensitive areas with fragile ecosystems. These countries are the primary sources of carbon emission growth due to high energy and carbon intensities, encountering the sharp contradiction between economic development and environmental protection.^85^ For instance, Southeastern Asia has become a focal point for infrastructure development due to vast conservation areas and biodiversity.^70^ However, the rapid development of the manufacturing sector has made it the fastest region in carbon emissions, threatening its ecological integrity. Thus, BRI countries should focus more on carbon emission mitigation while maintaining economic growth.

As a response, China proposed the “Green Belt and Road Initiative” in 2017 as an improved version of the BRI to support green development, aiming to achieve the Paris Agreement and promote SDGs.^86^ The decline in the proportion of carbon emissions provides strong evidence of the profound effects of China’s mitigation efforts in promoting green infrastructure investment and finance. Carbon emission mitigation has become a key issue in addressing climate change on a global scale. The implementation of emission trading systems^32^ and carbon taxes^34^ have been promoted as economically incentivized methods to advance carbon emission reduction. On the other hand, although the outbreak of COVID-19 has reduced global industrial activities and alleviated carbon emissions to some extent, the revival of economic activities has led to another expansion in the post-pandemic age.^24^ Regarding the implementation of nationally determined contributions under the Paris Agreement, while almost all BRI countries have put forward carbon emission reduction targets with specific implementation plans, there remains significant potential for reinforcing carbon mitigation strategies.^85^ In this context, the BRI is required to go through a green and low-carbon transition on an unprecedented scale. Based on the results and analysis, this study proposes several policy recommendations for carbon emission mitigation as follows.

(1) It is crucial to formulate practical, safe, and effective mitigation strategies, with the consideration of the basic national conditions and specific needs of stakeholders. In this context, regional carbon reduction targets and designed policy frameworks need to accommodate the distinct characteristics of various countries. The BRI should explore and pursue a new growth pathway driven by scientific and technological innovation and energy revolution to reduce the socioeconomic costs of climate change, avoiding the high-carbon lock-in trap.
(2) Addressing carbon inequality is not only a matter of environmental justice but also a crucial step in achieving global carbon emission reduction goals. The BRI should expand the energy cooperation platform to facilitate collaboration in advanced clean energy technology and demonstration projects, thereby facilitating resource sharing and complementarity and narrowing developmental gaps between and within countries.
(3) All the countries should commit to the principle of common but differentiated responsibilities, aligning their carbon reduction targets and actions based on their capacities. Developed countries are positioned to assist developing and emerging economies through technological support and financial aid. This cooperation model extends beyond mere assistance, which embodies a partnership aimed at harnessing sustainable technologies and practices that are crucial for an equitable and low-carbon future.

### Limitations of the study

This study focuses on an analysis of trade-embodied carbon emissions across 66 BRI countries from 2015 to 2019. Given the narrowly defined research regions, this study might not fully capture the complexities and heterogeneities across the global economy, especially those within the rapidly evolving BRI countries. An expansion of the geographical scope to a broader set of countries could provide a more comprehensive understanding of trade-embodied carbon flows and more nuanced mitigation strategies. Moreover, the time frame in this study excludes the recent shifts in global trade dynamics and the COVID-19 pandemic’s impacts on the global economy. An extension of the research period would enable an in-depth exploration of these pivotal changes and their potential impacts on carbon emission mitigation.

## STAR★Methods

### Key resources table

**Table:** 

REAGENT or RESOURCE	SOURCE	IDENTIFIER
**Deposited data**		

Global MREIO tables	Carbon Emission Accounts & Datasets (CEADs)	https://www.ceads.net.cn/data/input_output_tables
Data related to efficiency evaluation	The World Inequality Database (WID)	https://wid.world/data/
	The United Nations Statistics Division (UNSD)	https://unstats.un.org/UNSDWebsite
	The International Labor Organization (ILO)	https://ilostat.ilo.org/data
	The World Development Indicators (WDI)	https://databank.worldbank.org/source/world-development-indicators
Data related to empirical analysis	The World Development Indicators (WDI)	https://databank.worldbank.org/source/world-development-indicators

**Software and algorithms**		

ArcGIS 10.2	ESRI	https://www.arcgis.com/index.html
MATLAB 2018a	MathWorks	https://www.mathworks.com/products/matlab.html
Origin 2022b	OriginLab	https://www.originlab.com/index.aspx
StataSE 15	Statacorp	https://www.stata.com/products/index.html

### Resource availability

#### Lead contact

Further information and requests for resources and reagents should be directed to and will be fulfilled by the lead contact, Yung-Ho Chiu (echiu@scu.edu.tw).

#### Materials availability

The study did not generate new materials.

#### Data and code availability

• The sources of the datasets used in this study are all available from public resources, which are listed in the key resources table. Relevant data reported in this paper will be shared by the lead contact upon request.
• All custom code in this study written in MATLAB and Stata can be available from the lead contact upon reasonable request. This paper does not report original code.
• Any additional information required to reanalyze the data reported in this paper is available from the lead contact upon request.

### Method details

#### Data sources and collection

This study focuses on an aggregate level of BRI countries. Despite the extensive research on the BRI, the exact number and the list of the members remain ambiguous. In this study, a widely accepted geographical-based list of 66 BRI countries is applied,^5^^,^^20^^,^^87^ which stands as the most comprehensive investigation on the BRI constrained by data availability. In addition, CIS is treated as an individual region considering the unique economic attributes of itself.^88^ The detailed geographical classification of 66 BRI countries is listed in Table S3, with the abbreviations listed in Table S2.

The data used in this study can be categorized into three sections: (1) global MREIO tables for the accounting of trade-embodied carbon emissions; (2) input/output indicators for the efficiency evaluation of the manufacturing sector; (3) control variables for the empirical analysis.

First, the EMERGING database is adopted to track the latest trade-embodied carbon emissions in BRI countries. Whilst global MRIO databases (e.g., EXIOBASE, EORA, GTAP, GRAM, and WIOD databases) have rapidly emerged, the endeavors to account for carbon emissions have encountered stagnation due to the absence of timely and detailed high-resolution databases, especially for the emerging economies.^35^ The EMERGING database consists of nearly real-time and full-scale MREIO tables spanning from 2015 to 2019, which responds to this need powerfully. Second, six variables are selected as input/output indicators, which are referenced from the models constructed by Zhang and Wei in 2015^89^ and Feng et al. in 2017.^47^ Data sources used in this section include WID, UNSD, ILO, and WDI. Third, four variables are decided as control variables based on methodologies from relevant scholars,^82^^,^^83^^,^^90^ and core variables of empirical analysis are derived from the prior sections. The related data for this section primarily comes from WDI.

Notably, to align with the temporal coverage of the EMERGING database, the results and analysis in this study are based on this timeframe. This study further aggregates the 135 sectors of the database into 8 major sectors for convenience. The detailed sectoral aggregations can be found in Table S4. Economic indicators have been normalized to current US dollars based on global exchange rates. For certain missing data, the interpolation method has been employed to supplement all variables of the datasets. The specific definitions for the above indicators and variables are provided in Table S5.

#### MREIO analysis for accounting trade-embodied carbon emissions

This study calculates the trade-embodied carbon emissions within BRI countries based on the MREIO analysis framework. Suppose that there are m countries and n sectors in a country. Let i and j denote any country from the set of countries (1≤i,j≤m); r and s denote any sector from the set of sectors (1≤r,s≤m). Let $x_{r}^{i}$, $z_{\text{rs}}^{\text{ij}}$ and $y_{r}^{\text{ij}}$ denote the output of sector r in the country i, the intermediate demand of the sector s in country j for the commodities produced by the sector r in the country i and the final demand of the country j for the commodities produced by sector r in the country i, respectively. For a sector r in the country i, the overall monetary balance can be written as:

(Equation 1) $$x_{r}^{i}=\sum_{j=1}^{m}\sum_{s=1}^{n}z_{\text{rs}}^{\text{ij}}+\sum_{j=1}^{m}y_{r}^{\text{ij}}$$

Equation 1 can be written as the matrix form, which is more common:

(Equation 2) X=Zi+Y

X is the mn×1 total output column vector, Z is the mn×mn intermediate matrix; $i={\left[1,1,\cdots ,1\right]}^{\prime }$ is the mn×1 column vector, and Y is the mn×1 final demand (i.e., household, government, and capital) column vector. Additionally, X can be decomposed into the sum of domestic demand and international demand, which can be further written as the sum of intermediate demand and final demand like the overall balance:

(Equation 3) $$\left\{ \begin{array}{l} X=X_{d}+X_{\text{tr}}\\ X_{d}=\underset{i=j}{Z}i+\underset{i=j}{Y}\\ X_{\text{tr}}=\underset{i\neq j}{Z}i+\underset{i\neq j}{Y} \end{array}\right.$$

where $X_{d}$ indicates the domestic demand, and $X_{\text{tr}}$ indicates and international demand. When i=j, Z and Y denote the intermediate and final commodities intended for domestic use, respectively. On the converse, they denote the intermediate and final commodities exported to other countries, respectively. The above-balanced relationship applies to any country on Earth, and BRI countries are by no means an exception. Based on the accounting methods proposed by relevant scholars,^12^^,^^13^^,^^35^ the total amount of sectoral carbon emissions in the country i can be estimated as follows:

(Equation 4) $$C_{i}=K_{i}{\left(I-A_{i}\right)}^{-1}Y_{i}$$

where $K_{i}$ is the carbon intensity in the country i, which denotes the carbon emissions generated by one unit of total output; $L_{i}={\left(I-A_{i}\right)}^{-1}$ is the Leontief inverse matrix, which denotes the complete demand induced by one unit of final product. Considering the bilateral trade within BRI countries, the trade-embodied carbon emissions transferred from country i to country j (i≠j) can be calculated as:

(Equation 5) $$C_{\text{ij}}^{\text{tr}}=\overset{\text{tr}}{\underset{\text{ij}}{K}}\overset{\text{tr}}{\underset{\text{ij}}{L}}\overset{\text{tr}}{\underset{\text{ij}}{Y}}$$

where $K_{\text{ij}}^{\text{tr}}$, $L_{\text{ij}}^{\text{tr}}$, and $Y_{\text{ij}}^{\text{tr}}$ are matrices obtained after excluding domestic demand, which represent the carbon intensity, the Leontief inverse matrix, and the final demand of the BRI trade from country i to country j, respectively. Due to the symmetry of the intermediate and final demand matrices, it is straightforward to derive the trade-embodied carbon emissions transferred from country j to country i (i≠j). By taking the difference between the two, the net trade-embodied carbon emissions can be obtained.

Drawing upon the above accounting outcomes, we can identify the key priorities through a comparative analysis of trade-embodied carbon emissions at the sectoral/national level. Each sector/country can be ranked based on the scale of its trade-embodied carbon emissions annually, with top-ranking sectors/countries exerting major influence across the BRI. According to this principle, the sector that ranked highest from 2015 to 2019 should be identified as the key sector, and the countries with the most frequent appearances among the top ten countries from 2015 to 2019 should be identified as the key countries.

#### Dynamic DDF-based DEA model for assessing the efficiency of the key sector

This study constructs a dynamic DDF-based DEA model to assess carbon emission efficiency of the key sector in BRI countries over a continuous period. In reference to the selection of the input-output indicators of Zhang and Wei in 2015^89^ and Feng et al. in 2017,^47^ this study deals with n $\text{DM}U_{j}$ (j=1,2,⋯,n) over t period (t=1,2,⋯,T) in the presence of a non-discretionary input. For the selected indicators, the carbon inequality (Ceq) is treated as the non-discretionary input; the energy consumption (Nrg) and population employed (Lab) of the manufacturing sector are taken as discretionary inputs; the value added (VA), proportion of electricity (Elec), and the carbon emissions embodied in exports ($CO_{2}^{\text{ex}}$) in the manufacturing sector are taken as discretionary outputs; the capital stock of the manufacturing sector (Cap) is treated as the carry-over variable. Additionally, $CO_{2}^{\text{ex}}$ is treated as an undesirable output.

Let $I^{\text{ND}}$, $I^{D}$, and $L^{D}$ be the number of non-discretionary inputs $i^{\text{ND}}$, discretionary inputs $i^{D}$, and discretionary outputs $l^{D}$, $i\in i^{D}\cup i^{\text{ND}}$, $i^{D}\cap i^{\text{ND}}=\emptyset$. In the production system of the key sector in BRI countries, $I^{\text{ND}}=1$, $i^{\text{ND}}=1$ for $x_{i^{\text{ND}}\text{jt}}\geq 0$ of Ceq; $I^{D}=2$, $i^{D}=1,2$ for $x_{i^{D}\text{jt}}\geq 0$ of Nrg and Lab, respectively; $L^{D}=3$, $l^{D}=1,2,3$ for $y_{l^{D}\text{jt}}\geq 0$ of VA, Elec, and $CO_{2}^{\text{ex}}$, respectively. Additionally, this study treats $CO_{2}^{\text{ex}}$ as an undesirable output. Let $z_{j\left(t,t+1\right)}^{C}\geq 0$ of Cap be the carry-over variable for $\text{DM}U_{j}$ from the period t to the period t+1.

The production possibility set is $P_{t}=\left\{\left(x_{t}^{D},x_{t}^{\text{ND}},y_{t}^{D},z_{j\left(t,t+1\right)}^{C}\right),t=1,2,\cdots ,T\right\}$. The inherent distinction between efficiency and effectiveness measures lies in the consideration of input constraints during the assessment of the potential to achieve desired outcomes.^91^ The constraints of the indicators of $\text{DM}U_{o}$ (o=1,2,⋯,n) $\in P_{t}$ in the case of a dynamic DDF-based DEA model are as follows:

(Equation 6) $$\left\{ \begin{array}{l} x_{\text{ot}}^{D}\geq \sum_{j=1}^{n}x_{\text{jt}}^{D}\lambda_{\text{jt}}\\ x_{\text{ot}}^{\text{ND}}\geq \sum_{j=1}^{n}x_{\text{jt}}^{\text{ND}}\lambda_{\text{jt}}\\ y_{\text{ot}}^{D}\leq \sum_{j=1}^{n}y_{\text{jt}}^{D}\lambda_{\text{jt}}\\ z_{o,t-1}^{C}\geq \sum_{j=1}^{n}z_{j,t-1}^{C}\lambda_{\text{jt}}\\ \left(1+\theta_{t}\right)x_{\text{ot}}^{D}\leq \sum_{j=1}^{n}x_{\text{jt}}^{D}\lambda_{\text{jt}}\\ \left(1+\theta_{t}\right)y_{\text{ot}}^{D}\leq \sum_{j=1}^{n}y_{\text{jt}}^{D}\lambda_{\text{jt}}\\ \left(1+\theta_{t}\right)z_{\text{ot}}^{C}\leq \sum_{j=1}^{n}z_{\text{jt}}^{C}\lambda_{\text{jt}}\\ \sum_{j=1}^{n}\lambda_{\text{jt}}=1\\ \sum_{j=1}^{n}\lambda_{j,t-1}z_{j,t}^{C}=\sum_{j=1}^{n}\lambda_{j,t}z_{j,t}^{C}\\ \forall j,t\;\text{for} \end{array}\right.$$

where $\lambda_{\text{jt}}\geq 0$ is the intensity vectors of $\text{DM}U_{j}$. Based on Wanke et al. in 2018^57^ and Zhang et al. in 2021,^53^ the overall efficiency of DMU limited by the constraints can be defined as:

(Equation 7) $$\rho^{\text{overall}}=\max\;\sum_{t=1}^{T}\sum_{j=1}^{n}w_{t}\theta_{\text{jt}}$$

where $w_{t}\geq 0$ is the weight of period t, and $\sum_{t=1}^{T}w_{t}=1$. The value of $\rho_{,}^{\text{overall}}$ is within [0,1]. When the efficiency is close to 1, indicating the DMU proximity to the production frontier, the corresponding country is considered to be efficient in resource utilization and trade-embodied carbon emissions reduction. Conversely, it suggests that the corresponding country is inefficient, with the potential for resource utilization improvement and carbon emission mitigation.

#### Empirical models for examining the deep relationships between efficiency, emissions, and carbon inequality

Existing studies have substantiated that enhancing efficiency is an effective pathway for carbon emission mitigation.^44^^,^^45^^,^^48^ Building upon this foundation, this study employs a fixed effects panel model to investigate whether improving the efficiency of the key sector is the pathway to mitigating global carbon emissions. Moreover, natural logarithmic transformations are applied to variables for empirical analysis to address the challenges of variable unit inconsistency and heteroskedasticity. Based on the relevant studies,^82^^,^^83^^,^^90^ we established the baseline regression model as follows:

(Equation 8) $$CO_{\text{2 it}}^{\text{tr}}=\alpha_{0}+\alpha_{1}\text{CE}E_{\text{it}}+\alpha_{2}\text{Con}s_{\text{it}}+\lambda_{i}+\mu_{t}+\varepsilon_{\text{it}}$$

where $CO_{\text{2 it}}^{\text{tr}}$ indicates the sum of carbon emissions embodied in imports and exports of the country i in period t, $\text{CE}E_{\text{it}}$ denotes the trade-embodied carbon emission efficiency of the country i in period t, $\text{Con}s_{\text{it}}$ represents the set of national control variables. With reference to Liu et al. in 2020^81^ and Xu and Zhong in 2023,^82^ the economic growth (GDP), industrial structure (Ind), health level (Hlt), and medical service level (Med) are selected as control variables. Additionally, $\lambda_{i}$, $\mu_{t}$, and $\varepsilon_{\text{it}}$ denote the national individual fixed effect, time fixed effect, and the random error term, respectively. $\alpha_{i}$ represents the undetermined coefficient for each variable, which $\alpha_{1}$ is the coefficient of core interest. If $\alpha_{1}$ is insignificant, thus efficiency has no true impact on trade-embodied carbon emissions. If $\alpha_{1}$ is significantly positive, this indicates that enhancing efficiency might lead to carbon leakage, which is contrary to our expectations. If $\alpha_{1}$ is significantly negative, this demonstrates that efficiency improvement can help reduce carbon emissions, thereby confirming our hypothesis.

It is evident that carbon inequality exacerbates carbon emissions and further negatively impacts efficiency.^63^^,^^92^^,^^93^ To explore the moderating role of carbon inequality in the relationship between efficiency and trade-embodied carbon emissions, we introduce the interaction term in the baseline model. The moderating effect model is constructed as follows:

(Equation 9) $$CO_{\text{2 it}}^{\text{tr}}=\beta_{0}+\beta_{1}\text{CE}E_{\text{it}}+\beta_{2}\text{CE}E_{\text{it}}\times \text{Ce}q_{\text{it}}+\beta_{3}\text{Con}s_{\text{it}}+\lambda_{i}+\mu_{t}+\varepsilon_{\text{it}}$$

where $\text{Ce}q_{\text{it}}$ indicates carbon inequality of country i in period t, $\text{CE}E_{\text{it}}\times \text{Ce}q_{\text{it}}$ denotes the interaction term between efficiency and carbon inequality. $\beta_{i}$ represents the undetermined coefficient for each variable, which $\beta_{2}$ denotes the moderating effect of carbon inequality. If $\beta_{2}$ is insignificant, thus carbon inequality fails to stand as a moderating role. If $\beta_{2}$ is significantly positive, this indicates carbon inequality has a significant positive moderating effect. Conversely, carbon inequality turns out to have a significant negative moderating role.

### Quantification and statistical analysis

All statistical and empirical analyses were conducted using Stata software, with the specific version information listed in the key resources table. The center and dispersion measures employed in this study included mean and standard errors, respectively. Statistical significance was defined at conventional levels (∗∗∗p<0.01, ∗∗p<0.05, ∗p<0.1). Detailed empirical methodologies including baseline regression and moderating effect models are presented in the method details. The results of empirical analysis, with precise standard errors and significant levels, are presented in Table 7.

The efficiency evaluation and empirical analysis required the exclusion of samples from three countries—Turkmenistan, Syria, and East Timor—due to data unavailability. Consequently, the sample size included in the efficiency evaluation and empirical analysis was 63, and the total number of observations was 315 in the timeframe of 5 years from 2015 to 2019. For the empirical analysis, logarithmic transformations were applied to the variable data to prevent the challenges of variable unit inconsistency and heteroskedasticity. In the heterogeneous analysis, a total of 7 observations were excluded from the dataset due to the role transitions between net importers and exporters of certain BRI countries.

## Supplemental information

Document S1. Tables S1‒S5
