# Supplementary material for: Deep mitigation for trade-embodied carbon emissions among the Belt and Road Initiative countries
Source: iScience. 2024 May 22;27(8):110054. doi: 10.1016/j.isci.2024.110054 (PMC11342201; doi:10.1016/j.isci.2024.110054)
Supplement: Document S1. Tables S1‒S5 [file mmc1.pdf]

iScience, Volume *27*

Supplemental information

Deep mitigation for trade-embodied carbon emissions among the Belt and Road Initiative countries

Lina Zhang, Weichao Zhao, Yung-ho Chiu, Li Zhang, Zhen Shi, and Changfeng Shi

Supplemental information

Table S1. Abbreviations of terms, related to Tables 1,2.

Abbreviation	Full name
BRI	The Belt and Road Initiative
EU	The European Union
BRIC	Brazil, Russia, India, and China
APEC	Asia-Pacific Economic Cooperation
OECD	The Organization of Economic Cooperation and Development
CIS	The Commonwealth of Independent States
SDGs	Sustainable Development Goals
MREIO	Multi-Region Environmental Input-Output Analysis
DEA	Data Envelopment Analysis
DMU	Decision-Making Unit
DDF	Directional Distance Function
CEADs	Carbon Emission Accounts & Datasets
WID	World Inequality Database
UNSD	United Nations Statistics Division
ILO	International Labor Organization
WDI	World Development Indicators

Table S2. Abbreviations of 66 BRI countries, related to Tables 3,4,6 and Figures 3,5,7.

Country	Abbreviation	Country	Abbreviation	Country	Abbreviation	Country	Abbreviation
Afghanistan	AFG	East Timor	TMP	Lebanon	LBN	Saudi Arabia	SAU
Albania	ALB	Egypt	EGY	Lithuania	LTU	Serbia	SER
Armenia	ARM	Estonia	EST	Macedonia	MKD	Singapore	SGP
Azerbaijan	AZE	Georgia	GEO	Malaysia	MYS	Slovakia	SVK
Bahrain	BHR	Greece	GRC	Maldives	MDV	Slovenia	SVN
Bangladesh	BGD	Hungary	HUN	Moldova	MDA	Sri Lanka	LKA
Belarus	BLR	India	IND	Mongolia	MNG	Syria	SYR
Bhutan	BTN	Indonesia	IDN	Montenegro	MNE	Tajikistan	TJK
Bosnia and Herzegovina	BIH	Iran	IRN	Myanmar	MMR	Thailand	THA
		Iraq	IRQ	Nepal	NPL	Turkey	TUR
Brunei	BRN	Israel	ISR	Oman	OMN	Turkmenistan	TKM
Bulgaria	BGR	Jordan	JOR	Pakistan	PAK	Ukraine	UKR
Cambodia	KHM	Kazakhstan	KAZ	Philippines	PHL	United Arab Emirates	ARE
China	CHN	Kuwait	KWT	Poland	POL		
Croatia	HRV	Kyrgyzstan	KGZ	Qatar	QAT	Uzbekistan	UZB
Cyprus	CYP	Laos	LAO	Romania	ROU	Vietnam	VNM
Czechia	CZE	Latvia	LVA	Russia	RUS	Yemen	YEM

Table S3. Regional classification of 66 BRI countries, related to Table 5 and Figures 1,2,4,5,7.

Region	Country
Central and Eastern Asia	China, Kazakhstan, Kyrgyzstan, Mongolia, Tajikistan, Turkmenistan, Uzbekistan
Southeastern Asia	Brunei, Cambodia, Indonesia, Laos, Malaysia, Myanmar, Philippines, Singapore, Thailand, Vietnam
Southern Asia	Afghanistan, Bangladesh, Bhutan, India, Maldives, Nepal, Pakistan, Sri Lanka, East Timor
Western Asia and Africa	Bahrain, Cyprus, Egypt, Greece, Iran, Iraq, Israel, Jordan, Kuwait, Lebanon, Oman, Qatar, Saudi Arabia, Syria, Turkey, United Arab Emirates, Yemen
Europe	Albania, Bulgaria, Bosnia and Herzegovina, Czechia, Estonia, Croatia, Hungary, Lithuania, Latvia, Macedonia, Montenegro, Poland, Romania, Serbia, Slovakia, Slovenia
CIS	Armenia, Azerbaijan, Belarus, Georgia, Moldova, Russia, Ukraine

Table S4. Origin 134 sectors and aggregated 8 sectors, related to Table 5 and Figures 1,4.

Code	Origin Sector	Aggregated Sector
1-15	Live animals; Meat and edible meat offal; Fish, crustaceans, mollusks, aquatic invertebrates; Dairy products, eggs, honey, edible animal product; Products of animal origin; Live trees, plants, bulbs, roots, cut flowers; Edible vegetables and certain roots and tubers; Edible fruit, nuts, peel of citrus fruit, melons; Coffee, tea, mate and spices; Cereals; Milling products, malt, starches, inulin, wheat glute; Oil seed, oleic fruits, grain, seed, fruit; Lac, gums, resins, vegetable saps and extracts; Vegetable plaiting materials, vegetable products; Animal, vegetable fats and oils, cleavage products.	Agriculture
16-22	Coal; oil; gas; Petroleum, coal products; Salt, sulfur, earth, stone, plaster, lime and cement; Ores, slag and ash; Mineral fuels, oils, distillation products.	Mining
23-101	Meat, fish and seafood food preparations.; Sugars and sugar confectionery; Cocoa and cocoa preparations; Cereal, flour, starch, milk preparations and products; Vegetable, fruit, nut, food	Manufacturing

	preparations; Miscellaneous edible preparations; Beverages, spirits and vinegar; Residues, wastes of food industry, animal fodder; Tobacco and manufactured tobacco substitutes; Raw hides and skins (other than foreskins) and leather; Articles of leather, animal gut, harness, travel good; Foreskins and artificial fur, manufactures thereof; Silk; Wool, animal hair, horsehair yarn and fabric thereof; Cotton; Vegetable textile fibers, paper yarn, woven fabric; manmade filaments; manmade staple fibers; Wadding, felt, nonwovens, yarns, twine, cordage; Carpets and other textile floor coverings; Special woven or tufted fabric, lace, tapestry; Impregnated, coated or laminated textile fabric; Knitted or crocheted fabric; Articles of apparel, accessories, knit or crochet; Articles of apparel, accessories, not knit or crochet; Other made textile articles, sets, worn clothing.; Footwear, gaiters and the like, parts thereof; Headgear and parts thereof; Umbrellas, walking-sticks, seat-sticks, whips; Bird skin, feathers, artificial flowers, human hair; Wood and articles of wood, wood charcoal; Cork and articles of cork; Manufactures of plaiting material, basketwork,.; Pulp of wood, fibrous cellulosic material, waste; Paper & paperboard, articles of pulp, paper and board; Printed books, newspapers, pictures; Inorganic chemicals, precious metal compound, isotope; Organic chemicals; Pharmaceutical products; Fertilizers; Tanning, dyeing extracts, tannins, derivs, pigments; Essential oils, perfumes, cosmetics, toiletries; Soaps, lubricants, waxes, candles, modelling pastes; Albuminoids, modified starches, glues, enzymes; Explosives, pyrotechnics, matches, pyrophores; Photographic or cinematographic goods; Miscellaneous chemical products; Plastics and articles thereof; Rubber and articles thereof; Stone, plaster, cement, asbestos, mica, articles; Ceramic products; Glass and glassware; Pearls, precious stones, metals, coins; Iron and steel; Articles of iron or steel; Copper and articles thereof; Nickel and articles thereof; Aluminum and articles thereof; Lead and articles thereof; Zinc and articles thereof; Tin and articles thereof; Other base metals, cermet, articles thereof; Tools, implements, cutlery, of base metal; Miscellaneous articles of base metal; Nuclear reactors, boilers, machinery; Electrical, electronic equipment; Railway, tramway locomotives, rolling stock; Vehicles other than railway, tramway; Aircraft, spacecraft, and parts thereof; Ships, boats and other floating structures; Optical, photo, technical, medical, apparatus; Clocks and watches and parts thereof; Musical instruments, parts and accessories; Arms and ammunition, parts and accessories thereof; Furniture, lighting, signs, prefabricated buildings; Toys, games, sports requisites; Miscellaneous manufactured articles; Works of art, collectors pieces and antiques; Commodities not specified according to kind.	
102-104	Electricity; Gas manufacture, distribution; Water collection, purification, and distribution.	Resource supply industry
105	Construction.	Construction
106-114	Sea transport; Air transport; Other modes of transport; Postal and courier services; Goods (travel); Local transport services; Telecommunications services; Computer services; Information services.	Distribution service
115-118	Manufacturing services on physical inputs owned by others; Maintenance and repair services; Accommodation services; Food-serving services.	Consumer service
119-134	Direct insurance; Pension and standardized guaranteed services; Financial services; real estate; Charges for the use of intellectual property; Research and development services; Professional and management consulting services; engineering; Waste treatment and de-pollution agricultural and mining services; Operating leasing services; Other business services; Audiovisual and related services; Health services; Education services; recreation & other services; Government goods and services.	Production and other service

Table S5. Nomenclatures for indicators and variables, related to STAR Methods.

Indicator/Variable	Explanation	Definition
Ceq	Carbon inequality	The ratio of the top 1% per capita carbon emissions to the bottom 50% per capita carbon emissions
Nrg	Energy consumption	The total energy consumption of the manufacturing sector
Lab	Population employed	The population employed in the manufacturing sector
VA	Value added	The value added in the manufacturing sector
Elec	Access to electricity (SDG 7.1)	The proportion of electricity in the manufacturing sector
Cap	Capital stock	The fixed capital consumption of the manufacturing sector
CO_2^{ex}	Carbon emissions	The carbon emissions embodied in exports
CO_2^{tr}	Carbon emissions	The sum of carbon emissions embodied in imports and exports
CEE	Efficiency	The trade-embodied carbon emission efficiency
GDP	Economic growth	The gross domestic product
Ind	Industry development	Proportion of value added of industry in GDP
Hlt	Healthy level	Life expectancy at birth
Med	Medical level	Domestic general government medical expenditure
